# Growth failure of very low birth weight infants during the first 3 years: A Korean neonatal network

**DOI:** 10.1371/journal.pone.0259080

**Published:** 2021-10-28

**Authors:** Joohee Lim, So Jin Yoon, Jeong Eun Shin, Jung Ho Han, Soon Min Lee, Ho Seon Eun, Min Soo Park, Kook In Park

**Affiliations:** Department of Pediatrics, Yonsei University College of Medicine, Seoul, Korea; Centre Hospitalier Universitaire Vaudois, FRANCE

## Abstract

We aimed to evaluate risk factors for growth failure in very low birth weight (VLBW) infants at 18–24 months of corrected age (follow-up1, FU1) and at 36 months of age (follow-up2, FU2). In this prospective cohort study, a total of 2,943 VLBW infants from the Korean Neonatal Network between 2013 and 2015 finished follow-up at FU1. Growth failure was defined as a z-score below -1.28. Multiple logistic regression was used to analyze risk factors for growth failure after dividing the infants into small for gestational age (SGA) and appropriate for gestational age (AGA) groups. Overall, 18.7% of infants were SGA at birth. Growth failure was present in 60.0% at discharge, 20.3% at FU1, and 35.2% at FU2. Among AGA infants, male sex, growth failure at discharge, periventricular leukomalacia, treatment of retinopathy of prematurity, ventriculoperitoneal shunt status and treatment of rehabilitation after discharge were independent risk factors for growth failure at FU1. Among SGA infants, lower birth weight, pregnancy-induced hypertension, and treatment of rehabilitation after discharge were independent risk factors for growth failure at FU1. Mean weight z-score graphs from birth to 36 month of age revealed significant differences between SGA and non-SGA and between VLBW infants and extremely low birth weight infants. Growth failure remains an issue, and VLBW infants with risk factors should be closely checked for growth and nutrition.

## Introduction

Optimum postnatal growth in early infancy is critical for lowering metabolic and chronic diseases later in life and improving neurodevelopment in preterm infants [1–3]. Although preterm survival without major morbidities has improved in recent years through rapid evolvement of treatment strategies, it is unclear whether or not growth rates are better. Postnatal growth failure is typically diagnosed when an infant weigh less than the 10th percentile or has a weight z-score below -1.28 at discharge or at a postmenstrual age of 36-40-weeks [4]. However, the definition of growth failure, along with an appropriate duration of concern for growth, is debated [5]. Notwithstanding, growth failure occurs in approximately 5% to 10% of children in primary care settings and 3% to 5% of those in referral settings [6].

Identifying potential medical, nutritional, developmental, and psychosocial factors contributing to growth failure could help with improving growth outcomes in preterm infants. During admission in the neonatal intensive care unit (NICU), more aggressive nutritional approaches have been found to reduce the incidence of postnatal growth failure, although many questions about the expected rate of growth remain unanswered [7]. To date, intrauterine growth retardation is thought to reflect a genetic component and placenta function, and extrauterine growth retardation appears to be related with nutritional deficits and morbidity during NICU care. Meanwhile, research has revealed that infection, neonatal respiratory distress syndrome, and inadequate nutrition are risk factors for inadequate weight gain [8]. Medical risk factors for poor weight gain in infancy include prematurity, intrauterine growth restriction, developmental delay, congenital anomalies, intrauterine exposures, anemia, and any medical condition that results in in-adequate nutrient intake such as feeding problems, increased metabolic rate, maldigestion, or malabsorption [9–11].

Compared to previous decades, the worldwide incidence of postnatal growth failure has improved, although it is still a concern. According to the Vermont Oxford Network, the incidence of postnatal growth failure at discharge has decreased from 64.5% to 50.3%, whereas severe postnatal growth failure defined as a body weight below the third percentile has decreased from 39.8% to 27.5% [12]. In China, the incidence of extrauterine growth failure at the age of 1 year was 40.9% in 284 patients [13]. The National Institute of Child Health and Human Development (NICHD) reported that 40% of patients had a weight, length, and head circumference less than 10th percentile at 18–22 months [14].

Size at term equivalent age according to weight percentile or Z-score may not be associated with worse neurodevelopmental outcomes [15, 16]. However, ages and sizes after discharge up to 2 to 24 months have been shown to be associated with various poor neurologic outcomes, including cognitive delay, behavior problems, and cerebral palsy [17–19]. NICU teams should recognize that postnatal growth failure is a serious morbidity that needs prevention. Therefore, investigating longitudinal growth outcomes in very low birth weight (VLBW) infants after discharge and examining potential risk factors for growth failure after discharge are necessary for improving the quality of medical care in VLBW infants. The aim of this study was to evaluate the risk factors of growth failure within the first 3 years. Accordingly, we analyzed associations of growth failure at discharge from the NICU, 18–24 months of corrected age (FU1), and 36 months of age (FU2) with possible perinatal and post-discharge risk factors.

## Materials and methods

Data were extracted from the Korean Neonatal Network (KNN) database, which comprises data collected prospectively from 89 units across Korea; the data accounts for the care of more than 70% of VLBW infants born in Korea. The KNN database provides maternal and neonatal data from birth to the last follow-up visit, and all data are scheduled and collected by trained neonatologists using a standardized manual of operating procedures to minimize differences between sites. FU1 is scheduled at 18–24 months of corrected age and FU2 is scheduled at 33–39 months of chronological age by neonatologist working at KNN registered hospital according to the distributed manual of operation.

**Fig 1:**
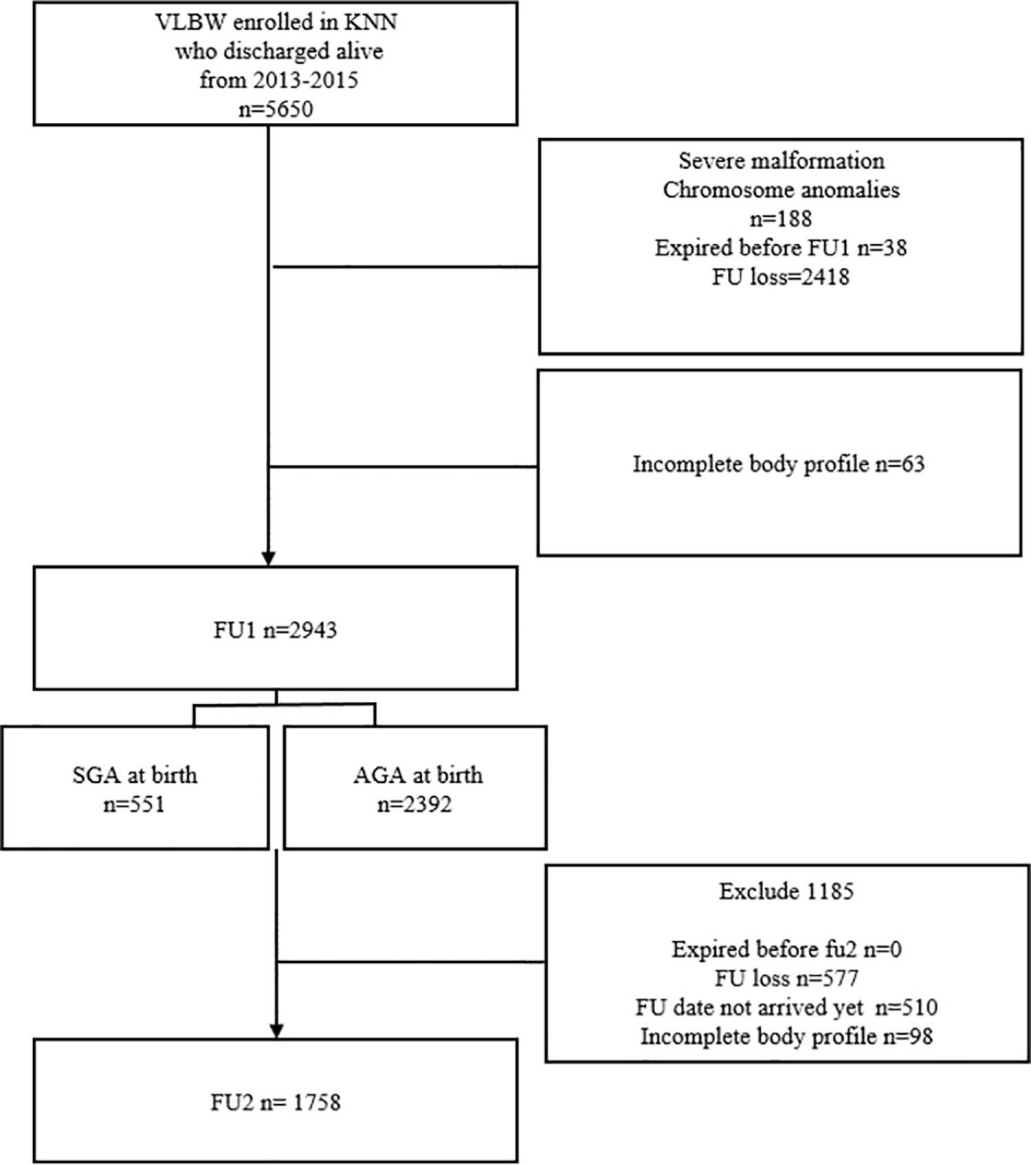
Flow chart of study population.

From 2013 to 2015, 5,650 VLBW infants had been discharged alive and were registered in the KNN. Among them, 188 infants with severe malformation or chromosome anomalies, 38 infants who expired within 18–24 months of age, 63 infants with inadequate body profile information without body weights, and 2418 infants who were lost to follow-up were excluded (Fig 1).

Demographic data, including the gestational age, birth weight, sex, maternal factors, comorbidities, and outcomes after discharge, were obtained from the database. Weight was measured following a standardized manual of operation from the KNN. Infant’s weight right after birth and at the time of discharge was measured on an electronic scale on a flat and hard surface. At FU1 and FU2, the infant stood on the scale in a neutral, still position with only a diaper or underwear. If they could not stand up, they were put on the scale in a supine position. the scale was zeroed before every measurement. Growth curves included the Fenton growth chart until a post-menstrual age of 50 weeks and the World Health Organization (WHO) growth charts at FU1 and FU2. Corrected age at FU1 and chronological age at FU2 was entered to an online WHO calculator to convert body weight to z-score (https://worldhealthorg.shinyapps.io/anthro/). Small for gestational age (SGA) was defined as a birth weight z-score below -1.28 (10th percentile) for gestational age using Fenton growth charts. Since, there is no international consensus regarding growth failure or catch-up growth of preterm infants, we defined growth failure as a weight below 10th percentile, severe growth failure as weight below the 3^rd^ percentile at discharge and follow up visits in this article.

The definitions of collected data were guided by the manual of operations of the KNN. Gestational age was determined from the obstetric history based on the last menstrual period. Antenatal steroid use was defined as the administration of any corticosteroid to the mother at any time before delivery to accelerate fetal lung maturity. Chorioamnionitis was confirmed by placental pathology and PROM was defined as rupture of membranes over 24 hours before the onset of labor. Bronchopulmonary dysplasia (BPD) was defined as the use of supplemental oxygen or respiratory support at 36 weeks’ post-menstrual age, corresponding to moderate to severe BPD using the severity-based definition for BPD of the National Institutes of Health consensus [20]. Severe intravascular hemorrhage (IVH) was defined as grade 3 or 4 according to the Papile’s classification [21]. Periventricular leukomalacia (PVL) was defined as cystic PVL based on either head ultrasonography or cranial magnetic resonance imaging performed at ≥2 weeks of age. Necrotizing enterocolitis (NEC) was defined as ≥ stage 2b according to the modified Bell criteria. Retinopathy of prematurity (ROP) was defined as ≥stage 3 during the NICU admission according to an international committee for the classification of ROP. Sepsis was defined by a positive blood culture in symptomatic infants suggestive of septicemia and more than 5 days of antibiotic treatment. Rehabilitation was defined as physical therapy to improve motor development.

All data are expressed as a mean ± standard deviation. Unadjusted comparisons between the two groups were performed using the chi-square or Fisher’s exact test for categorical data and the t-test for continuous data. Logistic regression was used to estimate the ORs with 95% CIs to identify the risk factor related with growth failure stratified by SGA and AGA group. In multiple logistic regression with backward elimination method, significantly different clinical factors associated with growth failure among SGA infants and AGA infants were identified. Models included covariates for perinatal factors, co-morbidities, and factors after discharge with p-value <0.05 in univariate analysis. A *P*-value < 0.05 was considered statistically significant. Statistical analyses were performed using SPSS version 21 (IBM Corp., Chicago, IL, USA). The level of statistical significance was set at *P* < 0.05.

The data registry was approved by the Samsung Medical Center Institutional Review Board (2013-03-002) and the institutional review boards of all 70 hospitals participating in the KNN. Written consent was obtained from the parents of infants during enrollment in the KNN. Data availability was subjected to the Act on Bioethics and Safety [Law No. 1518, article 18 (Provision of Personal Information)]. Contact for sharing the data or accessing the data can be possible only through the data committee of Korean neonatal network (http://knn.or.kr) and after permitted by the CDC of Korea. Detail contact information was as follows: data access committee: Yun Sil Chang (executive director of Korean neonatal network and sub-director of data and monitoring committee of Korean neonatal network, yschang@samsung.com) and ethics committee: Jang Hoon Lee (subdirector of ethics and publication committee of Korean neonatal network, neopedlee@gmail.com).

## Results

A total of 2,943 VLBW infants were included in the final analysis at FU1. At FU2, 1758 infants were included, as 577 infants were lost to follow-up, the follow-up date of 510 infants had not arrived yet, and 98 infants had lacking body weights. Infants who were lost to follow up showed similar baseline clinical characteristics, comparable to the follow-up group except for GA and BW (S1 Table). However, though the difference between the two groups were statistically significant, this was largely due to large sample size, and does not reflect a clinically relevant difference. The difference between the groups in rate of BPD may be more clinically relevant, but it is unclear whether it reflects a significant bias.

The 2,943 infants who completed follow-up at FU1 comprised 1498 (50.9%) boys, with an average gestational age of 28.8 weeks. The average birth weight was 1,091 g, with 36.4% of infants having extremely low birth weight. At birth, 19.4% of infants were SGA, with an average z-score of -0.30. Approximately 64% of infants were singleton, whereas 76.7% were born via c-section.

**Fig 2:**
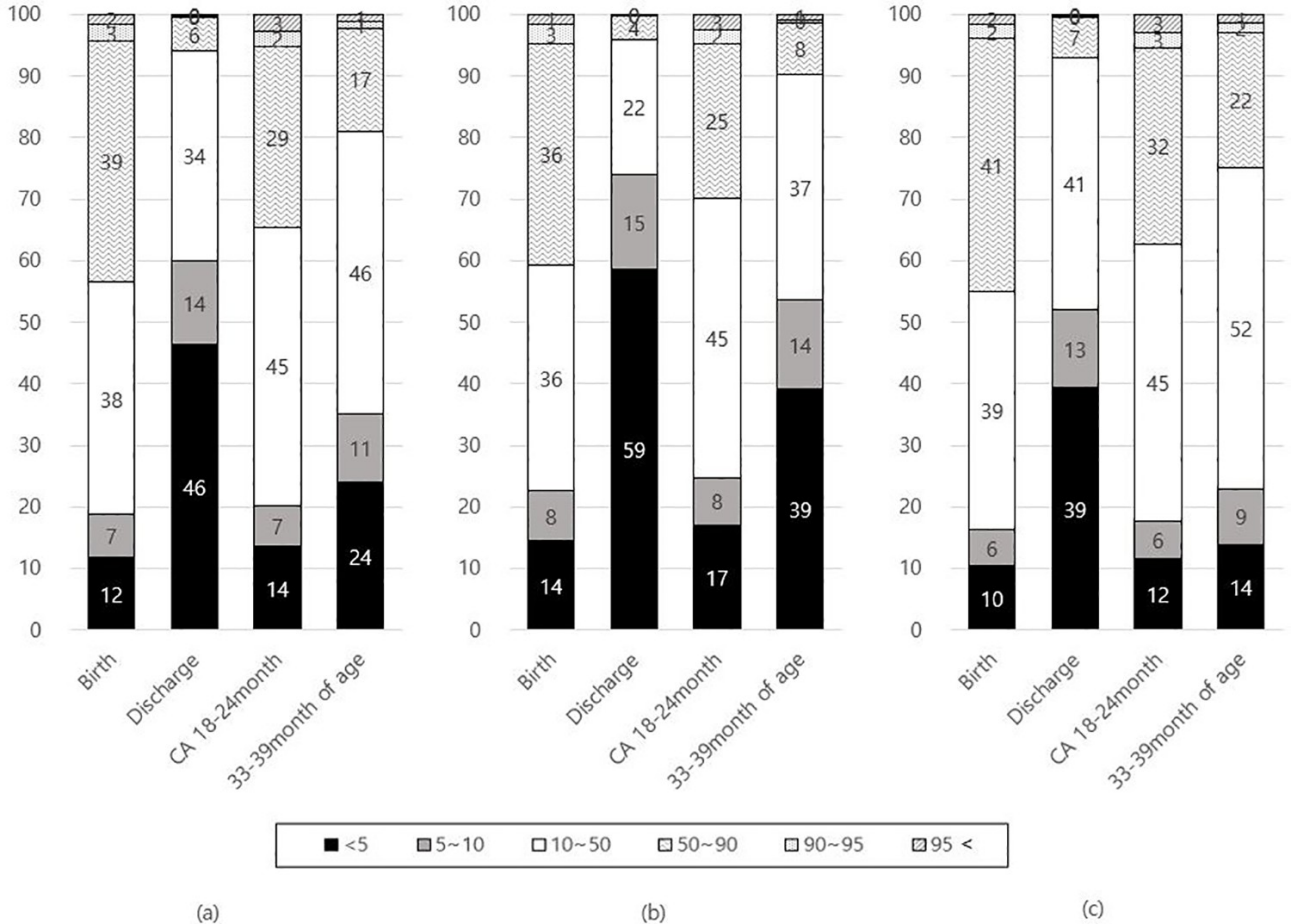
Proportions of weight percentiles at birth, discharge, follow-up 1, and follow-up 2 according to birth weight among VLBW infants (a), ELBW infants (b), and the infants with birth weight between 1000 and 1499g (c).

As seen in Fig 2, percent of infants below the 10th percentile was 19.4% at birth. At discharge, the average weight z-score was -1.4, with 60.0% exhibiting growth failure. At FU1, the average weight z-score was -0.47, with 21.9% exhibiting growth failure. Based on partial data at FU2, the rate of growth failure was 35.2%. The proportions of weight percentile among ELBW infants and the infants between 1000 and 1499g were shown respectively.

Table 1 showed the comparisons of perinatal factors, neonatal factors during NICU admission and post discharge factors between growth failure and normal growth group at FU 1 and FU 2. Because SGA status at birth was confirmed as a significant risk factor for the growth failure, we analyzed the risk factors for SGA and AGA group separately.

**Table 1:** Comparison of various factors between growth failure and normal growth at follow-up 1 and follow-up 2 by univariate analysis.

	Follow-up 1 (N = 2943)			Follow-up 2 (N = 1758)		
	Growth failure	Normal growth	P-value	Growth failure	Normal growth	P-value
	N = 545	N = 2398		N = 625	N = 1133	
**Perinatal factors**						
Gestational age, week	28.1±2.97	28.9±2.5	0.02	28.2± 2.8	28.9± 2.5	<0.01
Birth weight, g	927±278	1129±245	<0.01	931± 269	1141±241	<0.01
Male (%)	309 (56.7)	1190 (49.6)	<0.01	333 (53.3)	587 (51.8)	0.56
Caesarean delivery (%)	452 (82.9)	1806(75.3)	<0.01	499 (79.8)	824 (7.3)	<0.01
Multiple gestation (%)	192 (35.2)	860 (29.2)	0.78	233 (37.3)	442 (39.0)	0.48
IVF (%)	120 (22.0)	557(23.2)	0.57	167 (26.7)	299 (26.4)	0.88
Apgar score at 1min	4.3±2.0	4.7±1.9	<0.01	4.1± 2.0	4.7±2.0	0.85
Apgar score at 5min	6.6±1.7	6.9±1.7	<0.01	6.4± 1.9	6.9 ±1.72	<0.01
Rupture of membrane (%)	148 (14.0)	907 (86.0)	<0.01	204 (32.6)	439 (38.7)	0.02
Chorioamnionitis (%)	153 (32.6)	739 (35.3)	0.26	190 (30.4)	391 (34.5)	0.03
PIH (%)	113(20.7)	477 (19.9)	0.68	12 (1.92)	205 (18.1)	0.76
Maternal diabetes (%)	37 (6.8)	183 (7.6)	0.53	35 (5.6)	84 (7.4)	0.28
Prenatal steroid (%)	439 (80.5)	1908 (79.6)	0.54	333 (53.3)	549 (48.4)	0.15
SGA (%)	175(32.1)	397(16.6)	<0.01	163 (26.1)	199 (17.6)	<0.01
**Neonatal factors**						
Growth failure at discharge (%)	285 (52.29)	1394 (58.13)	0.01	405 (64.8)	593 (52.3)	<0.01
Resuscitation (%)	497 (91.7)	2122(89.1)	0.07	581 (92.9)	986 (92.9)	<0.01
HMD (%)	444 (81.5)	1905 (79.4)	0.31	527 (84.3)	876 (77.3)	<0.01
BPD(≥moderate) (%)	244 (44.9)	662 (27.6)	<0.01	278 (44.9)	318 (28.1)	<0.01
PDA ligation (%)	874 (3.0)	252 (8.7)	<0.01	111 (17.8)	111 (9.8)	<0.01
IVH (≥grade 3) (%)	70 (2.4)	118 (4.0)	<0.01	71 (11.4)	50 (4.4)	<0.01
PVL (%)	131(24.0)	432 (14.7)	<0.01	67 (10.7)	52 (4.6)	<0.01
NEC operation (%)	27 (61.3)	51 (56.0)	0.58	35 (5.6)	24 (2.1)	<0.01
Sepsis (%)	131 (4.5)	432 (14.7)	<0.01	136 (21.8)	185 (16.3)	<0.01
Idiopathic perforation (%)	14 (2.6)	31 (1.3)	0.03	17 (2.7)	12 (1.0)	<0.01
ROP treatment (%)	122(22.3)	237(9.8)	<0.01	200 (32.0)	178(15.7)	<0.01
**Post discharge factors**						
**Growth failure at FU1 (%)**	-	-	-	330 (52.8)	19 (1.7)	<0.01
Oxygen use (%)	100(18.7)	298(12.6)	<0.01	29 (4.6)	39 (3.4)	0.11
VP shunt (%)	35(6.6)	22(0.9)	<0.01	24 (3.8)	4 (0.35)	<0.01
Both parent as care giver (%)	406 (18.9)	1745(81.1)	0.51	495 (79.2)	860(75.9)	0.12
Attendance of day-care (%)	124(26.8)	786 (38.3)	<0.01	390 (62.4)	860 (75.9)	<0.01
Rehabilitation (%)	289 (54.4)	742(31.4)	0.62	214 (34.2)	206 (18.2)	<0.01
Readmission (%)	310 (58.9)	1083 (46.3)	<0.01	202 (32.3)	295 (26.0)	<0.01
Ophthalmologic treatment (%)	118(23.0)	307(13.6)	<0.01	120 (19.2)	118(10.4)	<0.01
Mechanical ventilator (%)	30(5.6)	108(4.6)	0.31	15 (2.4)	27 (2.4)	0.25
Tracheostomy (%)	8(1.5)	9(0.38)	<0.01	7 (1.1)	2 (0.2)	<0.01
Tube feeding (%)	65(12.2)	152(6.4)	<0.01	21 (3.4)	19 (1.7)	0.02

Values are expressed as numbers (%) and means ± standard deviations.

Abbreviations: IVF, in vitro fertilization; PIH, pregnancy-induced hypertension; SGA, small for gestational age; HMD, hyaline membrane disease; BPD, bronchopulmonary dysplasia: PDA, patent ductus arteriosus; IVH, intravascular hemorrhage; PVL, periventricular leukomalacia; NEC, necrotizing enterocolitis; ROP, retinopathy of prematurity; VP shunt, ventriculoperitoneal shunt.

Among 545 SGA infants, 207 infants (38%) showed growth failure which were associated for the following in univariate analysis: gestational age, birth weight, Apgar score, pregnancy-induced hypertension, hyaline membrane disease, BPD, Sepsis, ROP treatment, rehabilitation, readmission, oxygen use after discharge and attendance in daycare. (Table 2) However, multiple logistic regression analysis with backward elimination showed birth weight, pregnancy-induced hypertension and rehabilitation as independent risk factors for growth failure among SGA infants at FU1 (Table 3).

**Table 2:** Comparison of various factors between growth failure and normal growth groups at follow-up 1 in SGA infants and AGA infants by univariate analysis.

	Small for gestational age (N = 551)			Adequate for gestational age(N = 2392)		
	Growth failure	Normal growth	P-value	Growth failure	Normal growth	P-value
	N = 207	N = 344		N = 338	N = 2054	
Perinatal factors						
**Gestational age, week**	30.6±2.8	31.8±2.4	<0.01	27.4±2.3	28.4±2.1	<0.01
Birth weight, g	884±304	1113±278	<0.01	954±259	1132±240	<0.01
Male (%)	117 (56.5)	167 (48.6)	0.08	192 (56.8)	1023 (49.8)	0.02
Caesarean delivery (%)	194 (93.7)	314 (91.3)	0.330	258 (76.3)	1492 (72.6)	0.17
Multiple gestation (%)	68 (32.9)	97 (28.2)	0.25	124 (36.7)	763 (37.2)	0.90
IVF (%)	48 (23.2)	69 (20.1)	0.39	72 (21.3)	488 (23.8)	0.37
Apgar score at 1min	4.8±2.1	5.4±2.0	<0.01	4.0±1.9	4.6±1.9	<0.01
Apgar score at 5min	7.0±1.7	7.5±1.7	<0.01	6.4±1.7	6.8±1.7	<0.01
Rupture of membrane (%)	19 (9.2)	42 (12.2)	0.33	129 (38.2)	865 (42.1)	0.19
Chorioamnionitis (%)	38 (18.4)	52 (15.1)	0.55	115 (34.0)	687 (33.5)	0.43
PIH (%)	80 (38.7)	199 (57.9)	<0.01	47 (13.9)	322 (15.7)	0.46
Maternal diabetes (%)	16 (7.7)	27 (7.9)	1.00	26 (7.7)	180 (8.8)	0.60
Prenatal steroid (%)	439 (80.5)	1908 (79.6)	0.54	271 (80.2)	1658 (80.7)	1.00
**Neonatal factors**						
Growth failure at discharge (%)	200 (96.6)	334 (97.1)	0.23	249 (73.7)	975 (47.5)	<0.01
Resuscitation (%)	172 (83.1)	261 (75.9)	0.04	325 (96.2)	1861 (90.6)	<0.01
HMD (%)	131 (63.3)	169 (49.1)	<0.01	313 (92.6)	1736 (84.5)	<0.01
BPD(≥moderate) (%)	66 (31.9)	61 (17.7)	<0.01	180 (53.3)	603 (29.4)	<0.01
PDA ligation (%)	19 (9.2)	23 (6.7)	0.32	68 (20.1)	229 (11.2)	<0.01
IVH (≥grade 3) (%)	9 (4.4)	6 (1.7)	0.102	61 (18.1)	112 (5.5)	<0.01
PVL (%)	12 (5.8)	10 (2.9)	0.12	56 (16.6)	134 (6.5)	<0.01
NEC operation (%)	4 (1.9)	6 (1.7)	0.69	23 (6.8)	45 (2.2)	0.31
Sepsis (%)	40 (19.3)	39 (11.3)	0.01	91 (26.9)	393 (19.1)	<0.01
Idiopathic perforation (%)	5 (2.4)	5 (1.4)	0.51	9 (2.6)	26 (1.3)	0.08
ROP treatment (%)	49 (23.7)	26 (7.6)	<0.01	157 (46.5)	474 (23.1)	<0.01
**Post discharge factors**						
Oxygen use (%)	31 (15.0)	20 (5.8)	<0.01	69 (20.4)	278 (13.5)	<0.01
VP shunt (%)	2 (1.0)	1 (0.3)	0.56	33 (9.7)	21 (1.0)	<0.01
Both parent as care giver (%)	147 (71.0)	258 (75.0)	0.32	261 (77.2)	1507 (73.4)	0.14
Attendance of day-care (%)	51 (24.6)	119 (34.6)	<0.01	73 (21.6)	667 (32.5)	<0.01
Rehabilitation (%)	102 (49.3)	81 (23.6)	<0.01	187 (55.3)	661 (32.2)	<0.01
Readmission (%)	119 (57.5)	150 (43.6)	<0.01	191 (56.5)	933 (45.4)	<0.01
Ophthalmologic treatment (%)	32 (15.5)	19 (5.5)	<0.01	86 (25.4)	288 (14.0)	<0.01
Mechanical ventilator (%)	10 (4.8)	12 (3.5)	0.50	20 (5.9)	96 (4.7)	0.34
Tracheostomy (%)	1 (0.5)	1 (0.3)	1.00	7 (2.1)	8 (0.4)	<0.01
Tube feeding (%)	20 (9.7)	23 (6.7)	0.25	45 (13.3)	129 (6.3)	<0.01

Abbreviations: IVF, in vitro fertilization; PIH, pregnancy-induced hypertension; SGA, small for gestational age; HMD, hyaline membrane disease; BPD, bronchopulmonary dysplasia: PDA, patent ductus arteriosus; IVH, intravascular hemorrhage; PVL, periventricular leukomalacia; NEC, necrotizing enterocolitis; ROP, retinopathy of prematurity; VP shunt, ventriculoperitoneal shunt.

**Table 3:** Independent risk factors for growth failure at follow-up 1 among the infants with small for gestational age and appropriate for gestational age analyzed by multivariate analysis.

SGA infants (N = 551)			AGA infants (N = 2,392)		
	OR (95% CI)	p-value		OR (95% CI)	p-value
Birthweight	0.998 (0.997–0.998)	<0.01	male	1.531 (1.156–2.026)	<0.01
PIH	0.529 (0.343–0.816)	<0.01	Growth failure at discharge	2.437 (1.798–3.302)	<0.01
Rehabilitation	2.076 (1.315–3.276)	<0.01	PVL	1.847 (1.180–2.893)	<0.01
			ROP	1.425 (1.025–1.980)	<0.01
			VP SHUNT	6.359 (2.874–14.074)	<0.01
			Rehabilitation	1.462 (1.088–1.963)	0.01
			Birthweight	0.998 (0.998–0.999)	<0.01

*Odds Ratio Estimates and Wald Confidence Intervals

Abbreviations: PIH, pregnancy-induced hypertension; PVL, periventricular leukomalacia; ROP, retinopathy of prematurity; VP shunt, ventriculoperitoneal shunt.

Among 2,398 AGA infants, only 388 infants (14%) showed growth failure which were associated for the following in univariate analysis: male sex, gestational age, birth weight, Apgar score, growth failure at discharge, resuscitation, hyaline membrane disease, BPD, patent ductus arteriosus IVH, PVL, sepsis, ROP. In univariate analysis, Post discharge factors such as ventriculoperitoneal shunt, oxygen use, daycare attendance, rehabilitation and readmission, ophthalmologic treatment and tracheostomy, tube feeding were also associated. (Table 2). Using multiple logistic regression analysis with backward elimination, male sex, birth weight, growth failure at discharge, PVL, ROP, VP shunt, and rehabilitation were identified as independent risk factors for growth failure among AGA infants at FU1 (Table 3).

Among infants with growth failure at FU2, various prenatal factors such as gestational age, mode of delivery and 5 min Apgar score, ROM, chorioamnionitis, SGA were associated.

Morbidities during NICU, and related conditions until FU2 appeared to affect growth failure at FU2 in univariate analysis. Most of the morbidities during NICU stay were significantly associated with growth failure at FU2 (Table 1).

**Fig 3:**
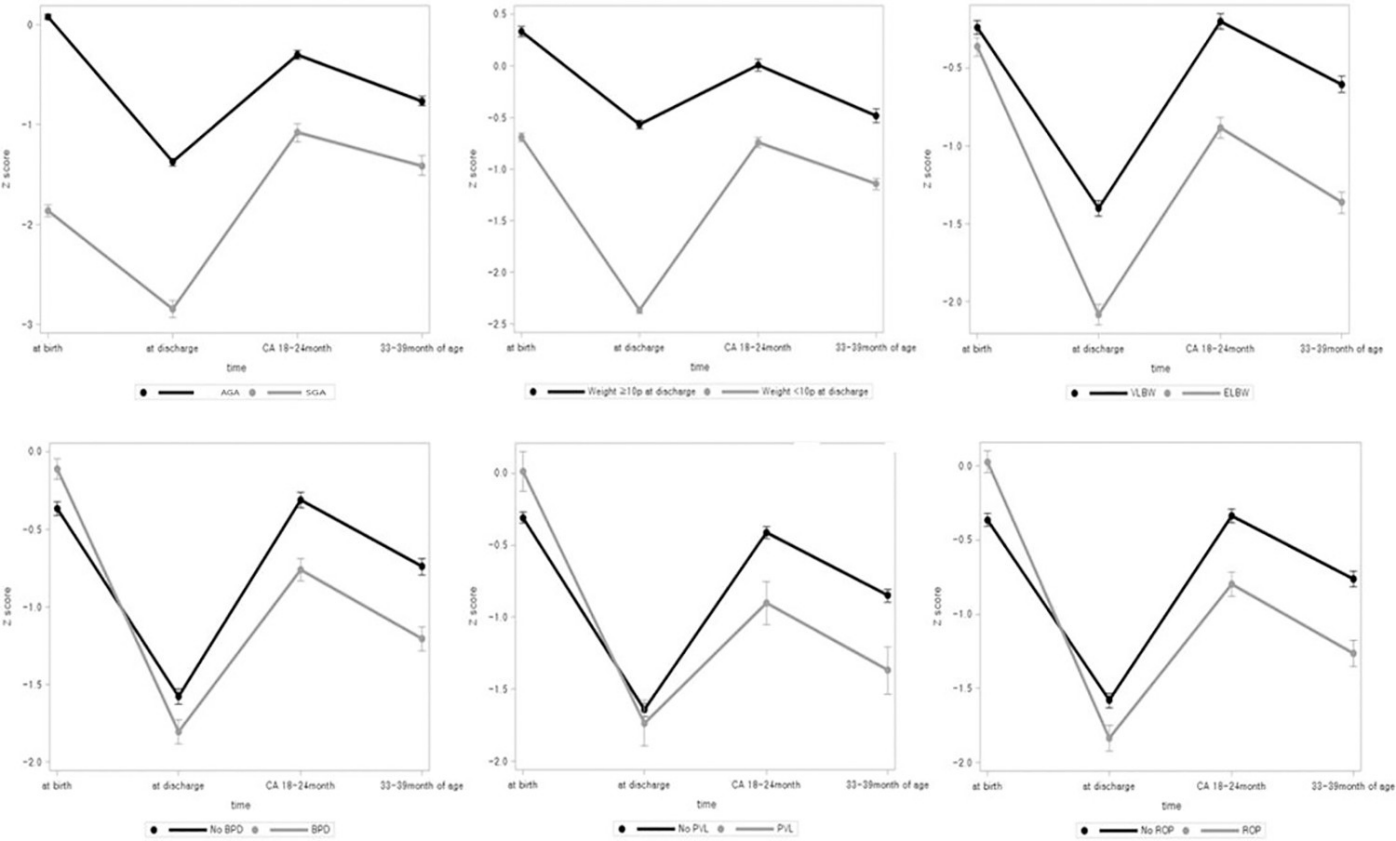
Mean weight z-score graph over time according SGA, growth failure at discharge, ELBW, BPD, PVL and ROP using a mixed model.

We analyzed the serial mean weight z-score over time using a mixed model in several factors (Fig 3). Whether infant is SGA at birth, growth failure at discharge, and growth failure at FU1 showed significant differences in serial z-score changes from birth to 3 years of age (p<0.0001). ELBW infants, the male infants, the infants with BPD, PVL, IVH and sepsis showed significant differences in serial z-score changes (p<0.0001).

## Discussion

Most preterm infants tend to achieve catch-up growth within the first 2 years of life [12], However, many studies have reported that catch-up growth can continue after infancy, even into adolescence [13–15]. This study confirmed that 20.3% of VLBW infants remained below the 10th percentile of weight at 18–24 months of postconceptional age and that 35.2% of VLBW infants remained below the 10th percentile of weight at 33–39 months. Further, growth after 3 years of age remained an issue. Therefore, it is important to analyze factors related to growth failure both during NICU care and after discharge for careful check-up and aggressive intervention.

Some studies argue that growth failure among premature infants is a physiological process [22]. At the time of discharge, preterm infants remain at an increased nutritional risk and require close growth monitoring after hospital discharge. Growth failure at discharge occurred in 60.0% of our study population, similar to other studies, such as 40.9% in China, 56.9% in India, and 50.3% in the Vermont Oxford Network [12, 23, 24]. Growth failure at the time of corrected age of 18–24 months occurred in 20.3% of infants, which is significantly less than that in data from the NICHD showing 40% of infants with weights, lengths, and head circumferences less than the 10th percentile at 18–22 months of corrected age [14]. Overall, our data indicate that advances in neonatal care in Korea have helped improve growth outcomes.

Previous studies do not agree on the time of onset of catch-up growth. A study in 2000 reported that 79% of 166 children below a birth weight of 1,000 g caught up weight before 3 years [25]. A recent study including 239 children showed that catch-up growth occurred mainly before discharge and extended until 6 years of age [26]. In contrast, a study in China showed that most preterm infants could achieve catch-up growth at a corrected age of 6 months [27]. Our data showed that infants who caught up at 18–24 months of postconceptional age could experience retarded growth again afterwards in the first 3 years, emphasizing close and serial follow-up for growth.

Growth is a complex process influenced by genetic, hormonal, and environmental factors [28]. From the KNN study, predictors of postnatal growth failure at discharge were respiratory distress syndrome and days to attain 100 mL/kg of enteral feeding in SGA infants and days to attain 100 mL/kg of enteral feeding only in AGA infants. From the study of VLBW infants in California, comorbidities most associated with poorer postnatal growth during NICU care were necrotizing enterocolitis, isolated gastrointestinal perforation, and severe retinopathy of prematurity. Most studies have focused on factors during NICU admission related to postnatal growth failure. However, few studies have analyzed factors associated with growth at 2–3 years of age, including condition after discharge from NICU. In this study, infants with oxygen use, tracheostomy, tube feeding, and ventriculoperitoneal shunt after discharge from NICU, readmission, and ophthalmologic condition with treatment were associated with growth failure at 18–24 months of age, in addition to comorbidities during NICU care and prenatal factors. Infants discharged with special care could be carefully monitored for growth.

The intrauterine environment plays a critical role in childhood growth. Distinctive growth patterns between SGA and non-SGA infants from birth to 3years of age were found in this study. We divided the infants by the SGA status for further analysis. Our study also emphasizes the weight below 10th percentiles at discharge and at corrected age of 18–24 month, showing the different weight patterns over time. It is very difficult to predict the ideal growth rate for a preterm newborn, careful monitoring of the growth rate over the three years of life is important.

Duration of NICU admission was relatively shorter than the post-discharge period until FU1, we found that independent risk factors for growth failure at FU1 in SGA infants were mainly pregnancy-induced hypertension, lower birth weight in addition to the rehabilitation treatment. These findings are similar to those of previous studies in which most children born to mothers with severe and early-onset hypertensive disorders of pregnancy were growth-restricted at birth and had complete catch-up growth at 4.5 years of age [29]. As rehabilitation can be speculated as another independent risk factor which may be related to growth failure, close follow up for development is also important.

As for AGA infants, male sex, lower birth weight, growth failure at discharge, and PVL, ROP, VP shunt status, rehabilitation treatment were confirmed as independent risk factors for growth failure at FU1. Thus, careful NICU care to reduce these morbidities during admission should be performed, and infants with known morbidities should undergo a regular check-up for growth monitoring.

Previous studies have indicated that infants with BPD face an increased risk of growth failure after hospital discharge with reported rates of 30% to 67%, similar to our findings [30]. This may be due to increased energy expenditure, reduced fat absorption, chronic hypoxia, and poor feeding performance [31, 32]. BPD (OR: 2.18) and the use of oxygen after discharge from the NICU (OR: 1.690) were significantly associated with growth at FU1; however, they were not independent risk factors in multiple regression. This may be due to restricted focus on weight gain rather than integrated growth including height and head circumference.

Our study provides valuable information of risk factors for growth failure in preterm infants. Efforts to promote growth should be made during admission and after discharge. Ongoing monitoring of growth is the first step in the management of growth failure. However, concerns for statistical growth cut-off values remain an issue, as excess nutrition delivery may also harm infants [4]. Further refinement of understanding of growth and nutrition is required.

A large cohort study including additional upcoming follow-up visits that can strengthen the results of the present study is being planned.

The limitation of this study is that our growth data included only weights at four time points (birth, discharge, FU1, and FU2) without considering height and head circumference or proportion, body composition, or genetic potential. The changes of weight and rate of growth is not considered. Data for FU2 is partial, as indicated earlier. We did not have detailed data on nutritional practices over the study period and, therefore, could not associate the changes in growth with specific changes in nutritional practice. Further studies should consider the type/nature of medical follow-up (other than the study visits), socioeconomic factors that could influence food availability, and parental feeding practices (type of milk, timing and nature of complementary foods), and parental education. Further study including weight gain velocity and change in weight z-score between timepoints would lead to more valuable results.

## Conclusions

Growth failure until 3 years of age remains an issue. Knowing perinatal history, reducing morbidity during admission, and performing regular check-ups after discharge is crucial in following up VLBW infants. Close follow-up to determine growth potential and to encourage proper intervention strategies should be performed for VLBW infants with risk factors. This study can lead to insight on growth in VLBW and offer intervention targets leading to well-being and proper growth in VLBW infants.

## Supporting information

S1 Table Comparison of clinical characteristics between follow up group and non-follow up group at follow-up 1. (DOCX)
