# Supplementary material for: Growth failure of very low birth weight infants during the first 3 years: A Korean neonatal network
Source: PLoS One. 2021 Oct 28;16(10):e0259080. doi: 10.1371/journal.pone.0259080 (PMC8553165; doi:10.1371/journal.pone.0259080)
Supplement: S1 Table — (DOCX) [file pone.0259080.s001.docx]

**S1 Table. Comparison of clinical characteristics between follow up group and non-follow up group at follow -up 1**

|  | Non follow up n=2481 | Follow up  n=2943 | p-value |
| --- | --- | --- | --- |
| Perinatal factors | | | |
| Gestational age, week | 28.5±2.9 | 28.8±2.6 | 0.00 |
| Birth weight, g | 1061.5±290.0 | 1091.9±264.0 | 0.00 |
| Male (%) | 1627 (65.6) | 1503 (51.1) | 0.20 |
| Caesarean delivery (%) | 1883 (75.8) | 2258 (76.7) | 0.48 |
| Multiple gestation (%) | 854 (34.4) | 1052 (35.7) | 0.31 |
| IVF (%) | 530 (21.4) | 677 (23.0) | 0.15 |
| Rupture of membrane (%) | 906 (36.5) | 1055 (35.8) | 0.58 |
| Chorioamnionitis (%) | 647 (26.0) | 892 (30.3) | 0.19 |
| Maternal diabetes (%) | 181 (7.3) | 220 (7.5) | 0.79 |
| Neonatal factors | | | |
| Resuscitation (%) | 2213 (89.2) | 2619 (89.0) | 0.61 |
| HMD (%) | 2028 (81.7) | 2349 (79.8) | 0.07 |
| BPD(≥moderate) (%) | 516 (20.8) | 906 (30.8) | 0.01 |
| PDA treatment (%) | 939 (37.8) | 1143 (38.8) | 0.46 |
| NEC operation (%) | 119 (4.8) | 78 (2.7) | 0.29 |

Values are expressed as numbers (%) and means ± standard deviations.

Abbreviations: IVF, in vitro fertilization; hyaline membrane disease; BPD, bronchopulmonary dysplasia: PDA, patent ductus arteriosus; NEC, necrotizing enterocolitis
